# Comparison between Intensity-Modulated Radiotherapy and Three-Dimensional Conformal Radiotherapy for Their Effectiveness in Esophageal Cancer Treatment: A Retrospective Single Institution Study

**DOI:** 10.1155/2020/6582341

**Published:** 2020-03-20

**Authors:** Xing-hua Bai, Jun Dang, Zhi-qin Chen, Zheng He, Guang Li

**Affiliations:** Department of Radiation Oncology, The First Affiliated Hospital of China Medical University, No. 155 Nanjing North Street, Shenyang, Liaoning 110001, China

## Abstract

Although a large number of influential studies that have been conducted worldwide on locally advanced esophageal cancer (EC) have employed the treatment modality of three-dimensional conformal radiotherapy (3D-CRT), an advanced as well as highly conformal technology known as intensity-modulated radiotherapy (IMRT) has attracted increasing attention from the radiotherapy research community. This is because of the clear advantages of IMRT, including decrease in radiation dose that reaches critical cardiopulmonary organs. These two treatment modalities need to be investigated with regard to their effect on local control rate and patient survival. In addition, related clinical factors also need to be explored. Data from a total of 431 patients with locally advanced EC, who underwent radiation therapy between January 1, 2010 and December 31, 2013, were included in the present study. Two hundred and ninety-three patients received 3D-CRT, while 138 patients received IMRT. We constructed propensity score matches to make the two groups be comparable (136 patients in 3D-CRT group and 138 patients in IMRT group. Kaplan–Meier analysis was conducted to evaluate the endpoint of overall survival (OS). A Cox proportional hazards model was employed to analyze the relationship between the associated factors and the outcomes via univariate and multivariate approaches. The mean follow-up period was 36.2 months, and the median follow-up period was 23 months. For the IMRT group, the median OS was 31 months, and the 1-, 3-, and 5-year OS rates were 70.3%, 50.0%, and 42.8%, respectively, while for the 3D-CRT group, the median OS was 22 months, and the 1-, 3-, and 5-year OS rates were 63.2%, 41.0%, and 35.4%, respectively (*p* < 0.05). The univariate analysis revealed that quit drinking, chemotherapy, and concurrent chemotherapy were significant risk factors for the prognosis of EC (*p* < 0.05), as well as the radiation therapy technique used (*p*=0.052). The multivariate analysis indicated that chemotherapy and quit drinking were independent predictive factors for OS. OS is found to be significantly better in the IMRT group, compared with that of the 3D-CRT group. Even though these outcomes need further validation, IMRT should be considered preferentially as a therapeutic option for EC, in combination with chemotherapy and persuading patients to quit drinking.

## 1. Introduction

It is reported that 572,034 new esophageal cancer (EC) cases have been diagnosed worldwide during 2018, with EC being the eighth most common malignancy and the sixth most likely cause of cancer-related deaths [1]. However, EC is difficult to be identified during its early stages. Additionally, patients often present with locally advanced stages or suffer from metastatic conditions at the time of diagnosis. Thus, although surgery is recognized as the most effective method of treatment, chemotherapy (CT) and radiotherapy (RT) have progressively become common and reliable modalities for EC treatment [2].

Two-dimensional (2D) treatment planning is accepted as the standard of care in the past. Later, CT-based three-dimensional (3D) treatment planning enhances target delimitation for the avoidance of normal structures, since it offers better anatomic visualization. Nevertheless, considerable doses are still received by normal tissues due to absence of dose variation for each of the three to five beams used for treatment. Along with the development of contemporary techniques, intensity-modulated radiation therapy (IMRT) uses linear accelerators to manipulate the photon beams of radiation to conform to the shape of a tumor, in order to securely, as well as painlessly deliver exact radiation doses to a tumor, while decreasing the dose delivered to adjacent normal tissues. The distinct dose-metric advantages of IMRT have been proven by several studies [3–6]. IMRT is costlier to implement and is also logistically more challenging, from treatment planning to the physics quality assurance procedure. Therefore, due to the limited scientific data to support the supremacy of IMRT, 3D conformal radiotherapy (3D-CRT) techniques have been extensively recognized as the present standard of care.

No differences in survival of the patients have been reported in two randomized trials conducted on chemoradiotherapy (CRT), with and without surgery [7, 8]. CRT is a viable option for EC treatment [9]. However, almost all seminal clinical trials for both CRT are based on 3D-CRT [10, 11]. Therefore, the advanced and highly conformal techniques of IMRT have been investigated only in a very limited clinical capacity [6, 12–16]. In the present setting, IMRT was firstly used in esophageal cancer treatment in 2010. From then, until 2016, over 400 patients had been treated with IMRT plus simultaneous chemotherapy, with or without surgery.

The aim of our study is to evaluate the outcomes of esophageal cancer patients treated at a single institution in order to compare IMRT with 3D-CRT.

## 2. Materials and Methods

### 2.1. Study Setting

This is a hospital-based retrospective cohort study on EC cases admitted to the First Affiliated Hospital of China Medical University. This hospital is a reference hospital situated in the northeastern region of China, with annual clinic visits of around 3.3 million person-visits and annual hospital admissions of around 15,000 person-admissions. The Department of Radiation Oncology accommodates approximately 6,500 cancer patient admissions each year, with about 150 of these patients being esophageal cancer patients.

### 2.2. Data Collection

Data of all patients who received radiation therapy for a diagnosis of nonmetastatic esophageal cancer between January 1, 2010, and December 31, 2013, were included in the present study. The corresponding characteristics of patients and the radiotherapy information were collected. After radiotherapy, patients' follow-up was scheduled every 6 months. Follow-up information was collected via telephone calls, outpatient clinic visits, and status check on the Census register. The end date of follow-up was September 7, 2017. The primary endpoint was overall survival (OS), which was defined as the time from esophageal cancer diagnosis to death. If the patient was alive at the end date of our study, the end date was adopted in the calculation. Patients who were alive or who died of other diseases were recorded as censored data.

Regarding lifestyle information, smokers or drinkers were defined as those who used that particular product for at least 6 months and reached the base level during this period (smoked at least 1 cigarette every 3 days/drank alcohol at least once a week). Smoking/drinking quitters were defined as those who had stopped smoking tobacco or consuming alcohol before the index date. Smoking/drinking duration was defined as the time between the age at which smoking/drinking began and either permanently stopping or the index date, after deducting the collective period of any episodes of provisionally quitting. Smoking intensity was defined as the mean number of cigarettes smoked each day. Alcohol consumption was defined as the mean volume of ethanol consumed each day. Criteria for the risk of intake was based on WHO grouping, and are as follows [17]: low risk (male: 1 to 40 g; female: 1 to 20 g), medium risk (male: 41 to 60 g; females: 21 to 40 g), high risk (male: 61 to 100 g; female: (41 to 60 g), very high risk (male: ≧101 g; female: ≧61 g).

### 2.3. Statistical Analysis

The chi-square test was carried out to analyze the patient characteristics. Propensity score matches were used to get two comparable groups. Survival analysis was conducted using the Kaplan–Meier methodology. Cox proportional hazards modeling was employed to analyze factors associated with the outcomes using univariate and multivariate approaches. A *p* value of <0.05 was considered to be statistically significant. SPSS (IBM, Shanghai, version 21.0) was used for analysis.

## 3. Results

### 3.1. Patient Characteristics

A total of 543 patients with esophageal cancer received treatment at the hospital from 2010 to 2013. The patient exclusion criteria included (1) patients who did not receive radiation therapy or whose information was duplicated (*n* = 96); (2) patients who did not have primary esophageal cancer (*n* = 8); (3) patient survival information was proved wrong (*n* = 5); and (4) patients who underwent two types of radiotherapy (*n* = 3). Finally, the data of 431 patients who received radiotherapy were retrospectively reviewed in the current study. Among these patients, 138 patients received IMRT, while 293 received 3D-CRT. A description of the common characteristics of the patients included is provided in Table 1.

Patients in IMRT group were less likely to quit cigarette smoking (IMRT, 10.7%; 3D-CRT, 21.9%; *p* < 0.05), more likely to receive chemotherapy (IMRT, 57.2%; 3D-CRT, 37.5%; *p* < 0.05), and more likely to receive previous treatment (IMRT, 45.7%; 3D-CRT, 31.4%; *p* < 0.05).

**Figure 1:**
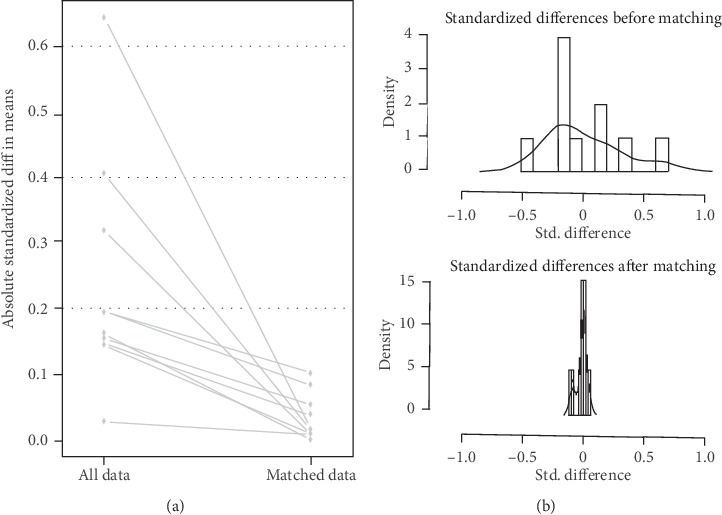
Effect of propensity score matches evaluating by absolute standardized differences and density of standardized differences.

To decrease the imbalances between the two groups, the propensity score matches was constructed in the factors of the patients' gender, age, smoking status, drinking status, weight loss, pathology type, quit smoking, chemotherapy, and previous treatment. The result of propensity score matches is showed in Table 2, and the effect of it is showed in Figure 1. After propensity score matches, we got two comparable groups (136 patients in 3D-CRT group and 138 patients in the IMRT group).

### 3.2. Outcomes of Therapy

**Figure 2:**
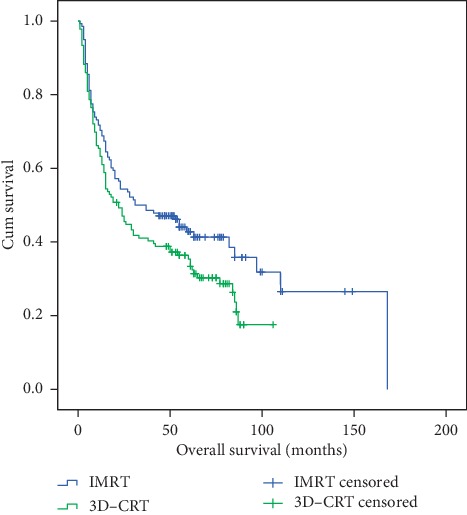
Overall survival of patients in the intensity-modulated radiotherapy (IMRT) and three-dimensional conformal radiotherapy (3D-CRT) groups. The 1-, 3-, and 5-year OS rates were 70.3%, 50.0%, and 42.8% (in IMRT group) vs. 63.2%, 41.0%, and 35.4% (in the 3D-CRT group) (*p* < 0.05).

In the IMRT group, the median OS was 31 months, while the 1-, 3-, and 5-year OS rates were 70.3%, 50.0%, and 42.8%, respectively, whereas in the 3D-CRT group, the median OS was 22 months, while the 1-, 3-, and 5-year OS rates were 63.2%, 41.0%, and 35.4%, respectively (*p* < 0.05) (Figure 2).

### 3.3. Prognostic Factors of Overall Survival

Patients' characteristics were assessed to recognize their predictive value for OS (Table 3). The univariate analysis found that quit drinking, chemotherapy, and concurrent chemotherapy were significant risk factors for the prognosis of EC (*p* < 0.05), as well as the radiation therapy technique used (*p*=0.052).

**Figure 3:**
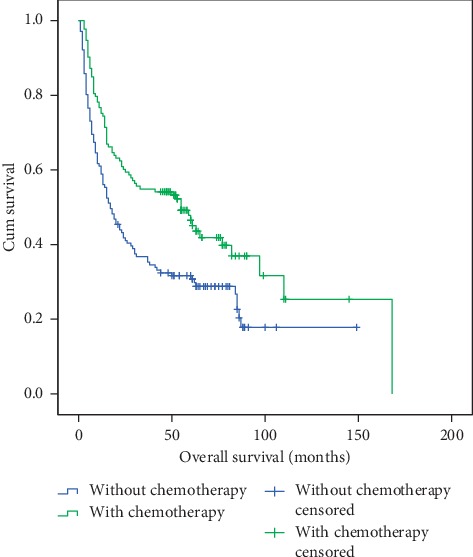
Overall survival of patients in the chemotherapy and without chemotherapy groups. The 1-, 3-, and 5-year OS rates were 75.2%, 54.9%, and 46.5% (in chemotherapy group) vs. 58.9%, 36.7%, and 31.7% (in the without chemotherapy group) (*p* < 0.05).

**Figure 4:**
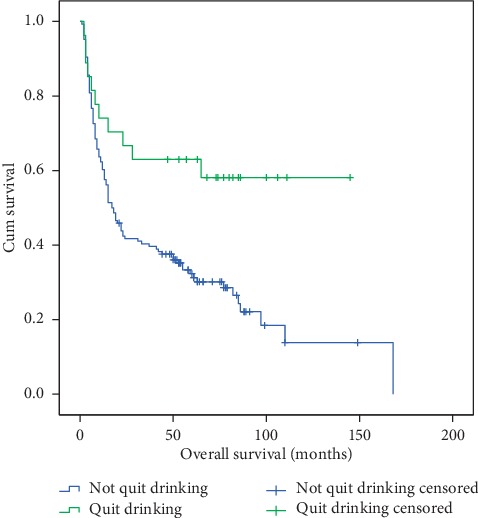
Overall survival of patients in the quit drinking and not quit drinking groups. The 1-, 3-, and 5-year OS rates were 74.1%, 63.0%, and 63.0% (in the quit drinking group) vs. 60.3%, 40.3%, and 32.3% (in the not quit drinking group) (*p* < 0.05).

These four factors were used for multivariate analysis, and the result revealed that chemotherapy and quit drinking were independent factors that influence EC OS (Table 4). The hazard ratios (HR) with 95% confidence interval (CI) were 0.477 (0.247–0.921) (*p*=0.027) and 0.395 (0.221–0.741) (*p*=0.004), respectively. In the “with chemotherapy” group, the median OS was 55 months, and the 1-, 3-, and 5-year OS rates were 75.2%, 54.9%, and 46.5%, respectively. However, in the “without chemotherapy” group, the median OS was 17 months, and the 1-, 3-, and 5-year OS rates were 58.9%, 36.7%, and 31.7%, respectively (*p* < 0.05) (Figure 3). In the “quit drinking” group, the median OS was 63 months, and the 1-, 3-, and 5-year OS rates were 74.1%, 63.0%, and 63.0%, respectively. However, in the “not quit drinking” group, the median OS was 17 months, and the 1-, 3-, and 5-year OS rates were 60.3%, 40.3%, and 32.3%, respectively (*p* < 0.05) (Figure 4).

## 4. Discussion

During the past three decades, key developments in surgery and radiotherapy, as well as chemotherapy had created multimodel tactics that could be used as therapeutic treatment alternatives for EC. For patients with unfeasible or irresectable conditions, definitive CRT is an optional choice of treatment.

Our study paid special attention to the variation in radiotherapy modalities from an overall survival point of view. IMRT develops target conformity and diminishes radiation dose to neighboring organs. However, its clinical benefits to patients with esophageal cancer are not well understood. The total economic cost has also been under debate. However, if this modality could decrease the number of perioperative complications, the worries regarding its economic burden may be offset.

There are several trials that evaluate the use of IMRT for the treatment of EC. The effectiveness and safety of IMRT for locally advanced esophageal squamous cell carcinoma (ESCC) had been evaluated by Ge et al. with the study having reported that IMRT was a practical and feasible technique for the treatment of ESCC [18]. Zhang et al. found that postoperative IMRT could reduce local recurrence, as well as improve survival of lymph node-positive or stage III thoracic esophageal squamous cell carcinoma (TESCC) patients [19].

In our study cohort, we compared the overall survival of patients treated with 3D-CRT with that of IMRT patients, using data from a large group of EC patients with long-term follow-up. We found that there was substantial enhancement in OS in patients of the IMRT group, compared with that of the 3D-CRT group. These results corresponded to previously published findings. A meta-analysis that compared IMRT and 3D-CRT for the treatment of EC found that IMRT was better than 3D-CRT in terms of overall survival of EC, while no difference with regard to radiation harmfulness was found [14]. A study carried out on 676 nonrandomized patients from 1998–2008 evaluated the effect of chemoradiotherapy on stage Ib-IVa EC patients, and indicated a significant improvement in OS, locoregional control, and noncancer-related death, when comparing IMRT with 3D-CRT [20]. IMRT had been reported to be superior to CRT for the treatment of upper EC along with simultaneous integrated boost (SIB) [5]. Lin et al. evaluated the therapeutic effect and toxicity of IMRT or 3D-CRT in 60 patients with locally advanced EC and found that IMRT appeared to be more effective than 3D-CRT [21]. On the other hand, there are several studies that have yielded ambiguous or contradictory results, as well. It had been reported that there was no significant difference in overall survival, failure-free survival, as well as regional failure-free survival, among the IMRT patients (*n* = 64) and 2D-RT patients (*n* = 37) for the treatment of cervical esophageal squamous cell carcinoma from 2001 to 2012 [15]. No substantial survival advantages were detected from the use of 3D-CRT/IMRT in a retrospective analysis based on four prospective clinical trials, which assessed the toxicity as well as long-term survival of ESCC patients treated with 3D-CRT or IMRT in comparison with that of 2D-RT [22]. The ambiguous or contradictory results may be due to the lack of data concerning the potential value of IMRT in a dose-escalated radiotherapy setting. The authors point out that this will become an important issue and challenge for the future, if the use of IMRT for EC gradually increases [23].

According to the univariate analysis results of the present study, the “without chemotherapy” was found to be a statistically significant risk factor for EC, showing that IMRT and chemotherapy could together prolong overall survival. A study of 170 patients with locally advanced EC from 2004 to 2008 reported similar results, indicating that IMRT with chemotherapy could decrease the local recurrence rate, prolong overall survival, and regression-free survival, but gave rise to more side effects [24].

Several limitations need to be acknowledged in the present study. The observational nature and relatively smaller sample size cannot be avoided. The effect of new supportive technologies, such as staging PET and 4-dimensional CT planning, cannot be overlooked too. Additionally, institutional biases in referring EC patients for radiotherapy also need to be considered.

In conclusion, we found that overall survival significantly improved with IMRT-treated, compared with that of 3D-CRT-treated esophageal cancer patients, but we should also pay special attention to the effect of chemotherapy, as well as change of lifestyle, such as quit drinking. Ideally, these outcomes should be analyzed using a larger randomized trial that compares these two modalities. Our results provide evidence of the potential of IMRT in improving outcomes than traditional treatment methods, and IMRT can be actively considered for use in esophageal cancer treatment, in combination with chemotherapy and persuading patients to quit drinking.

## Figures and Tables

**Table 1:** Characteristics of the EC patients grouped by the radiotherapy technique.

Characteristic	IMRT (*n* = 138)	3D-CRT (*n* = 293)	*p* value
Gender			0.094
Male	128 (92.8%)	256 (87.4%)	
Female	10 (7.2%)	37 (12.6%)	

Age (years)			0.209
<55	36 (26.1%)	63 (21.5%)	
55∼65	56 (40.6%)	107 (36.5%)	
65∼75	34 (24.6%)	76 (26.0%)	
≥75	12 (8.7%)	47 (16.0%)	

Type of medical insurance			0.307
None	30 (21.7%)	83 (28.3%)	
New rural cooperative medical insurance	27 (19.6%)	58 (19.8%)	
Urban employee or resident insurance	81 (58.7%)	152 (51.9%)	

Smoking status			0.078
No	26 (18.9%)	78 (26.6%)	
Yes	112 (81.1%)	215 (73.4%)	

Smoking intensity (cig/d)			0.331
1∼10	5 (4.5%)	22 (10.2%)	
11∼20	25 (22.3%)	41 (19.1%)	
21∼40	69 (61.6%)	128 (59.4%)	
>40	13 (11.6%)	24 (11.3%)	

Smoking duration (years)			0.331
<20	3 (2.7%)	13 (6.0%)	
20∼40	72 (64.3%)	126 (58.6%)	
≧40	37 (33.0%)	76 (35.4%)	

Quit smoking			0.013
No	100 (89.3%)	168 (78.1%)	
Yes	12 (10.7%)	47 (21.9%)	

Drinking status			0.793
No	50 (36.2%)	110 (37.5%)	
Yes	88 (63.8%)	183 (62.5%)	

Alcohol consumption			0.314
Low risk	22 (25.0%)	48 (26.2%)	
Medium risk	11 (12.5%)	23 (12.6%)	
High risk	33 (37.5%)	50 (27.3%)	
Very high risk	22 (25.0%)	62 (33.9%)	

Drinking duration (years)			0.094
<20	16 (18.2%)	30 (16.4%)	
20∼40	49 (55.7%)	100 (54.6%)	
>40	23 (26.1%)	53 (29.0%)	

Quit drinking			0.326
No	75 (85.2%)	147 (80.3%)	
Yes	13 (14.8%)	36 (19.7%)	

Weight loss (kg)			0.183
No	10 (7.2%)	29 (9.9%)	
<5	76 (55.1%)	184 (62.8%)	
≧5, <10	29 (21.0%)	40 (13.6%)	
≧10, <15	13 (9.5%)	27 (9.2%)	
≧15	10 (7.2%)	13 (4.5%)	

Pathology			0.121
Squamous cell carcinoma	120 (87.0%)	229 (78.1%)	
Adenocarcinoma	2 (1.4%)	16 (5.5%)	
Adenosquamous carcinoma	1 (0.7%)	4 (1.4%)	
Small-cell carcinoma	0 (0.0%)	4 (1.4%)	
Unknown	15 (10.9%)	40 (13.6%)	

Chemotherapy			0.0001
No	59 (42.8%)	183 (62.5%)	
Yes	79 (57.2%)	110 (37.5%)	

Concurrent chemotherapy			0.286
5-Fu + Platinum-based drugs	55 (70.5%)	68 (63.6%)	
Cisplatin	8 (10.3%)	20 (18.7%)	
Others	15 (19.2%)	19 (17.7%)	

Radiation dose (Gy)			0.171
<60	55 (39.9%)	97 (33.1%)	
≧60	83 (60.1%)	196 (66.9%)	

Previous treatment			0.004
No	75 (54.3%)	201 (68.6%)	
Yes	63 (45.7%)	92 (31.4%)	

Previous treatment methods			0.001
Surgery	36 (57.1%)	67 (72.8%)	
Chemotherapy	5 (7.9%)	16 (17.4%)	
Radiation therapy	16 (25.5%)	8 (8.7%)	
Chemoradiotherapy	6 (9.5%)	1 (1.1%)	

EC, esophageal cancer; IMRT, intensity-modulated radiation therapy; 3D-CRT, three-dimensional conformal radiation therapy; kg, kilogram; Gy, gray unit.

**Table 2:** The result of propensity score matches of all patients.

Subsamples	All		Matched		Unmatched		Discarded	
	Control	Treated	Control	Treated	Control	Treated	Control	Treated
All cases	138	293	138	136	0	157	0	0

**Table 3:** Univariate analysis of prognostic factors for EC patients' overall survival.

Factors	Wald chi-square	HR	*p* value
Gender	0.097	1.107	0.751
Age	0.119	1.003	0.730
Type of medical insurance	1.483	1.120	0.218
Smoking status	0.043	1.040	0.835
Smoking intensity	0.002	0.995	0.965
Smoking duration	0.104	0.949	0.746
Quit smoking	0.256	0.890	0.608
Drinking status	0.740	1.145	0.387
Alcohol consumption	0.694	0.936	0.407
Drinking duration	0.193	1.073	0.660
Quit drinking	7.323	0.422	0.002
Weight loss	0.338	0.967	0.559
Pathology	0.334	0.964	0.556
Chemotherapy	10.584	0.610	0.001
Concurrent chemotherapy	3.776	0.836	0.044
Radiation therapy techniques	3.740	1.339	0.052
Radiation dose	1.684	0.819	0.198
Previous treatment	0.129	0.947	0.719
Previous treatment methods	0.000	0.999	0.991

EC, esophageal cancer.

**Table 4:** Multivariate analysis of prognostic factors for EC patients' overall survival.

Factors	Chi-square	HR	95% CI	*p* value
Chemotherapy	4.867	0.477	0.247–0.921	0.027
Quit drinking	8.378	0.395	0.221–0.741	0.004

EC, esophageal cancer.

## Data Availability

The data that support the findings of this study are available from the corresponding author on reasonable request.
